# Arctigenin from *Arctium lappa *inhibits interleukin-2 and interferon gene expression in primary human T lymphocytes

**DOI:** 10.1186/1749-8546-6-12

**Published:** 2011-03-25

**Authors:** Wei-Jern Tsai, Chu-Ting Chang, Guei-Jane Wang, Tzong-Huei Lee, Shwu-Fen Chang, Shao-Chun Lu, Yuh-Chi Kuo

**Affiliations:** 1 National Research Institute of Chinese Medicine, Taipei, 11221, Taiwan; 2 Institute of Life Science, Fu-Jen University, Taipei, 24205, Taiwan; 3 Graduate Institute of Pharmacology Science, Taipei Medical University, Taipei, 11031, Taiwan; 4 Graduate Institute of Medical Sciences, Taipei Medical University, Taipei, 11031, Taiwan; 5 Department of Biochemistry and Molecular Biology, College of Medicine, National Taiwan University, Taipei, 10051, Taiwan

## Abstract

**Background:**

*Arctium lappa *(*Niubang*), a Chinese herbal medicine, is used to treat tissue inflammation. This study investigates the effects of arctigenin (AC), isolated from *A. lappa*, on anti-CD3/CD28 Ab-stimulated cell proliferation and cytokine gene expression in primary human T lymphocytes.

**Methods:**

Cell proliferation was determined with enzyme immunoassays and the tritiated thymidine uptake method. Cytokine production and gene expression were analyzed with reverse transcription-polymerase chain reaction.

**Results:**

AC inhibited primary human T lymphocytes proliferation activated by anti-CD3/CD28 Ab. Cell viability test indicated that the inhibitory effects of AC on primary human T lymphocyte proliferation were not due to direct cytotoxicity. AC suppressed interleukin-2 (IL-2) and interferon-γ (IFN-γ) production in a concentration-dependent manner. Furthermore, AC decreased the IL-2 and IFN-γ gene expression in primary human T lymphocytes induced by anti-CD3/CD28 Ab. Reporter gene analyses revealed that AC decreased NF-AT-mediated reporter gene expression.

**Conclusion:**

AC inhibited T lymphocyte proliferation and decreased the gene expression of IL-2, IFN-γ and NF-AT.

## Background

The central event in the generation of immune responses is the activation and clonal expansion of T cells. Interaction of T cells with antigens initiates a cascade of biochemical events and gene expression that induces the resting T cells to activate and proliferate [1]. Activation of nuclear factor of activated T cells (NF-AT) and a series of genes such as interleukin-2 (IL-2) and interferon-γ (IFN-γ) are pivotal in the growth of T lymphocytes induced by antigens [2,3]. Thus, growth modulators or other external events affecting T cell proliferation are likely to act by controlling the expression or function of the products of these genes [4]. The immune responses to invasive organisms, if inappropriately intense or prolonged, may paradoxically aggravate the injury or even cause death. The use of immunomodulatory medications must therefore be discreet. Regulation of T lymphocyte activation and proliferation and cytokine production is one of the action mechanisms [5,6].

Chinese medicinal herbs are now widely acknowledged for their immunomodulatory activities [1]. A member of the Compositae family, *Arctium lappa *(*Niubang*) is regarded as an effective Chinese medicine for alleviation of rheumatic pain and fever [7]. Arctigenin (AC), a bioactive component of *A. lappa*, has various biological activities including: (1) inhibition of nitric oxide, interlukin-6 and tumor necrosis factor-α production in macrophages [8,9]; (2) anti-proliferative activity against leukemia cells [10]; and (3) protective effects on hepatocytes from CCl_4 _injury [11]. Definitive evidence for its effects on T cell-mediated immune responses has been scarce.

The present study aims to elucidate the effects of AC on T lymphocytes proliferation, production and gene expression of IL-2 and IFN-γ in T lymphocytes induced by anti-CD3/CD28 antibodies (Ab) and NF-AT activation.

## Methods

### Preparation of arctigenin (AC)

**Figure 1:**
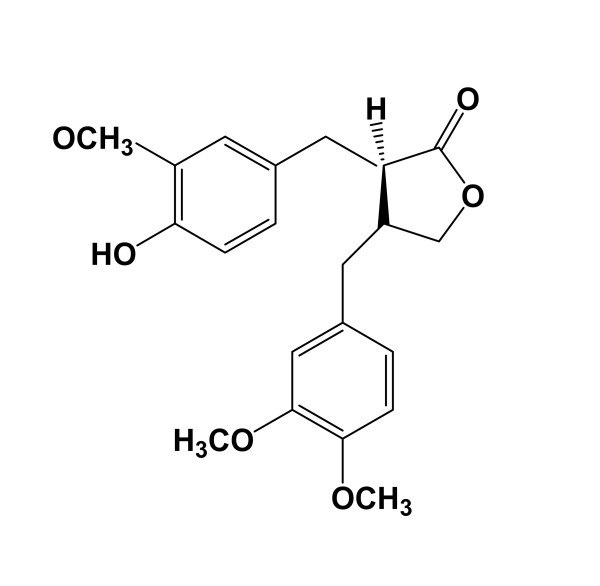
**Chemical structure of AC**.

AC was isolated from dried ground of *A. lappa *L. by using reported methods [12]. Briefly, ground *A. lappa *(1 kg) was extracted with ethanol (2L × 3) at room temperature. The solvent was removed under reduced pressure and the residue was partitioned between H_2_O and ethyl acetate (EtOAc). The concentrated EtOAc extracts (60 mg) were subjected to chromatography over silica gel and eluted with *n*-hexane/EtOAc (4:1), *n*-hexane/EtOAc (1:1) and EtOAc successively. AC (4.5 mg; C_21_H_24_O_6_; MW 372; Figure 1) was purified from EtOAC fraction with bioassay-guided separation. Mass and NMR spectral data for this compound were identical with those previously reported [12]. AC, with the purity above 98%, was dissolved in dimethylsulfoxide (DMSO) to a concentration of 100 mM and then stored at 4°C until use.

### Participants

Ten healthy male participants aged between 20 and 32 years (mean 26) were selected for this study. The experimental protocol were reviewed and approved by the institutional human experimentation committee of Fu-Jen University. Written informed consent was obtained from all participants.

### Preparation of primary human T lymphocytes

Heparinized human peripheral bloods (80 ml) were obtained from healthy donors. The peripheral blood was centrifuged at 850 × *g *(Sorvall Legend RT, Kendro, Germany) at 4°C for ten minutes to remove the plasma. The blood cells were diluted with phosphate buffered saline (PBS) and then centrifuged in a Ficoll-Hypaque discontinuous gradient (specific gravity 1.077) at 420 × *g *(Sorvall Legend RT, Kendro, Germany) for 30 minutes. The peripheral blood mononuclear cell (PBMC) layers were collected and washed with cold distilled water and 10× Hanks' buffer saline solution (HBSS) to remove red blood cells. T lymphocytes were separated from PBMC by nylon wool columns (Wako Chemicals, USA). Purified T lymphocytes had >87% CD3^+ ^cells and <0.5% CD14^+ ^or CD19^+ ^cells. The cells were re-suspended to a concentration of 2 × 10^6 ^cells/ml in RPMI-1640 medium supplemented with 2% fetal calf serum (FCS), 100 U/ml penicillin and 100 μg/ml streptomycin [4].

### Lymphoproliferation test

The lymphoproliferation test was modified from a previously described method [13]. Briefly, the density of T lymphocytes was adjusted to 2 × 10^6 ^cells/ml before use. Cell suspension (100 μl) was applied into each well of a 96-well flat-bottomed plate (Nunc 167008, Nunclon, Denmark) with or without anti-CD3 (1 μg/ml)/CD28 (3 μg/ml) antibody (eBioscience, USA). Cyclosporin A (CsA, 2.5 μM), an immuno-suppressor, was used as a reference drug [14]. AC was added to the cells at various concentrations (6.25, 12.5 and 25 μM). The plates were incubated in 5% CO_2_-air humidified atmosphere at 37°C for three days. Subsequently, tritiated thymidine (1 μCi/well, New England Nuclear, USA) was added into each well. After incubated for 16 hours, the cells were harvested on glass fiber filters by an automatic harvester (Dynatech, Multimash 2000, UK). Radioactivity (counting per minute, CPM) in the filters was measured by a scintillation counter (LS 6000IC, Beckman Instruments Inc., USA). The inhibitory activity of AC on T lymphocytes proliferation was calculated according to the following formula:

Inhibitory activity (%) = [Control group (CPM) - Experiment group (CPM)]/Control group (CPM) × 100%

### Determination of IL-2 and IFN-γ production

Primary human T lymphocytes (2 × 10^5 ^cells/well) were cultured with anti-CD3/CD28 Ab alone or in combination with cyclosporin A (CsA) or various concentrations of AC for three days. The cell supernatants were then collected and assayed for IL-2 and IFN-γ concentrations by enzyme immunoassays (EIAs; R&D systems, USA).

### Determination of cell viability

Resting or anti-CD3/CD28 Ab-activated T lymphocytes were cultured in a medium, namely DMSO (0.1%), or various concentrations of AC (6.25, 12.5 and 25 μM) for four days. After stained by trypan blue, total, viable and non-viable cell numbers were counted with a hemocytometer under microscope. The percentage of viable cells was calculated according to the following formula:

Viability (%) = (Viable Cell Number/Total Cell Number) × 100%

### Extraction of total cellular RNA

T lymphocytes (5 × 10^6^) were activated with or without anti-CD3/CD28 Ab and co-cultured with 6.25, 12.5 or 25 μM of AC for 18 hours. T lymphocytes were collected and lysed by RNA-Bee™ (Tel-Test, USA). After centrifugation with 12000 × *g *(Sigma 2K15, B Braun, Germany) at 4°C for 15 min, the supernatants were extracted with a phenol-chloroform mixture. The extracted RNA was precipitated with 100% cold ethanol. The total cellular RNA was pelleted by centrifugation and re-dissolved in diethyl pyrocarbonate (DEPC)-treated water. The concentration of RNA was calculated according to its optical density at 260 nm.

### Reverse transcription-polymerase chain reaction (RT-PCR)

RT-PCR was carried out according to a previously described method [15]. Briefly, RNA (1 μg) was reverse-transcribed to cDNA by the Advantage™ RT-for-PCR kit (Clontech, USA) according to the manufacturer's instructions. Briefly, 10 μl of cDNA was mixed with 0.75 μM primers, four units of Taq polymerase, 10 μl of reaction buffer consisting of 2 mM Tris-HCl (pH8.0), 0.01 mM ethylenediaminetetraacetate (EDTA), 0.1 mM dithiothreitol (DTT), 0.1% Triton X-100, 5% glycerol and 1.5 mM MgCl_2_, and 25 μl of water making up a total volume of 50 μl. All primer pairs for the glyceraldehyde-3-phosphate dehydrogenase (GAPDH), IL-2, and IFN-γ were designed according to the published human cDNA sequence data (Table 1). Settings of the PCR thermocycler were as follows: denaturing at 94°C for 1 minute, annealing at 60°C for 1 minute and elongation at 72°C for 80 seconds for the first 35 cycles and finally elongation at 72°C for 10 minutes. After the reaction, the amplified products were run on 1.8% agarose gel for electrophoresis.

**Table 1:** Oligonucleotide sequences of the primers used for amplification of IL-2, IFN-γ and GAPDH mRNA in primary human T lymphocytes

Cytokine	Sequence	Predicted size (bp)
IL-2	5'-GTC ACA AAC AGT GCA CCT AC-3' 5'-GAA AGT GAA TTC TGG GTC CC-3'	262
IFN-γ	5'-GCA GAG CCA AAT TGT CTC CT-3' 5'-ATG CTC TTC GAC CTC GAA AC-3'	320
GAPDH	5': TGA AGG TCG GAG TCA ACG GAT TTG GT 3': CAT GTG GGC CAT GAG GTC CAC CAC	983

### Luciferase assay

Jurkat cells (5 × 10^4^) were transfected by pGL4.30 (luc2P/NFAT-RE/Hygro) with Lipofectamin™ 2000 (Invitrogen, USA) for 24 hours according to the manufacturer's instructions. Then, the cells were cultured with anti-CD3 (1 μg/ml)/CD28 (3 μg/ml) Ab in the presence or absence of AC (6.25, 12.5 and 25 μM) or CsA (2.5 μM) for four hours. Total cell lysates were extracted with 1× reporter lysis buffer (Promega, USA). Total cell lysates (10 μg) were used to determine luciferase activity by the Luciferase Assay System (Promega, USA).

### Statistical analysis

Data were presented as mean ± standard deviation (SD). The differences between groups were assessed with student's *t *test and corrected with the Bonferroni test. Correlations between AC concentration and activity parameters were calculated with Pearson product-moment correlation test. *P *< 0.05 was considered statistically significant.

## Results

### Effects of AC on primary human T lymphocytes proliferation

**Figure 2:**
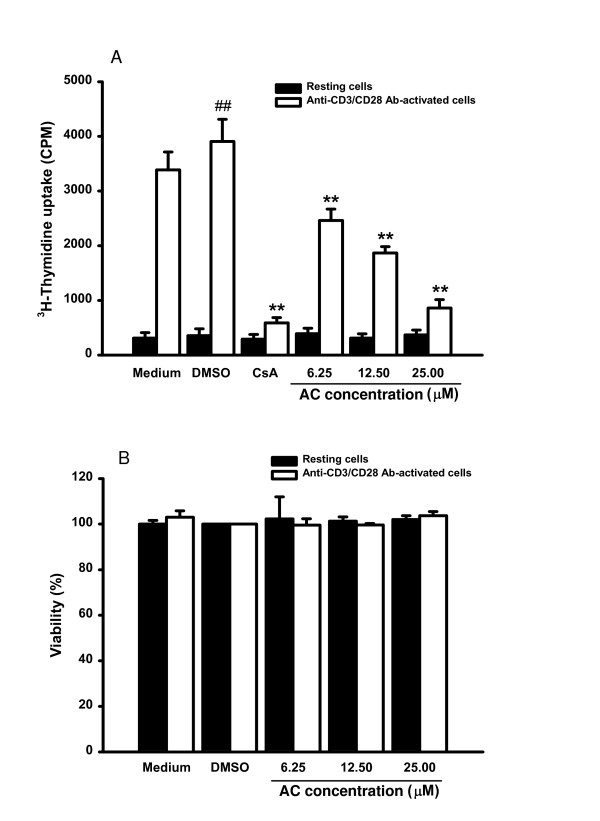
**Effects of AC on cell proliferation and cell viability in primary human T lymphocytes**. Primary human T lymphocytes (2 × 10^5^/well) were stimulated with or without anti-CD3 (1 μg/ml)/CD28 (3 μg/ml) Ab and treated with medium, 0.1%DMSO, or the indicated concentration of AC, or CsA (2.5 μM). (A) After incubated for 72 hours, the proliferation of T lymphocytes was detected by tritiated thymidine uptake (1 μCi/well). After incubated for 16 hours, the cells were harvested by an automatic harvester, then radioactivity was measured by liquid scintillation counting. (B) After 96 hr incubation, T cells were harvested and numbers of total, viable, and nonviable cells were counted after trypan blue staining. Each bar represents the mean ± SD of three independent experiments. ^## ^*P *< 0.01: vs. the cells treated with DMSO. ** *P *< 0.01: vs. the cells treated with DMSO and anti-CD3/CD28 Ab.

Using indicated concentrations of AC isolated from *A. lappa*, we treated resting cells or cells activated with anti-CD3/CD28 Ab. Cell proliferation was determined by tritiated thymidine uptake. As shown in Figure 2A, treatment with anti-CD3/CD28 Ab for three days increased cell proliferation by about 11-fold (*P *= 0.002). Neither the resting or the activated cells was affected by DMSO treatment in terms of the tritiated thymidine uptake. While AC had little effect on tritiated thymidine uptake in resting T lymphocytes, the enhanced uptake observed in the activated cells was significantly suppressed by 6.25, 12.5 and 25 μM AC (*P *= 0.006, *P *= 0.007 and *P *= 0.002 respectively). The inhibition of AC on the activated cells were in a dose-dependent manner (*r *= -0.963, *P *= 0.0374). At 6.25 μM, the inhibitory percentage of AC was 37.0 ± 5.0% on T lymphocytes proliferation activated by anti-CD3/CD28 Ab. The corresponding degree of inhibition for 12.5 μM was 52.1 ± 2.9% whereas that for 100 μM was 78.0 ± 4.0%. The IC_50 _of AC on activated primary human T lymphocytes proliferation was 15.7 ± 3.2 μM.

### Viability of primary human T lymphocytes treated with various concentrations of AC

We examined the viabilities of resting or anti-CD3/CD28 activated T lymphocytes treated with 6.25, 12.5 and 25 μM respectively for four days. AC had no cytotoxicity, *ie *the viabilities of resting or activated cells were not significantly decreased after treatment with various concentrations of AC for four days (Figure 2B). In comparison with the medium-treated group, neither the viability of the resting T lymphocytes nor that of the anti-CD3/CD28-activated T lymphocytes was reduced by DMSO, indicating that decreased T lymphocytes proliferation by AC was not related to direct cytotoxicity.

### Effects of AC on IL-2 and IFN-γ production in primary human T lymphocytes

**Figure 3:**
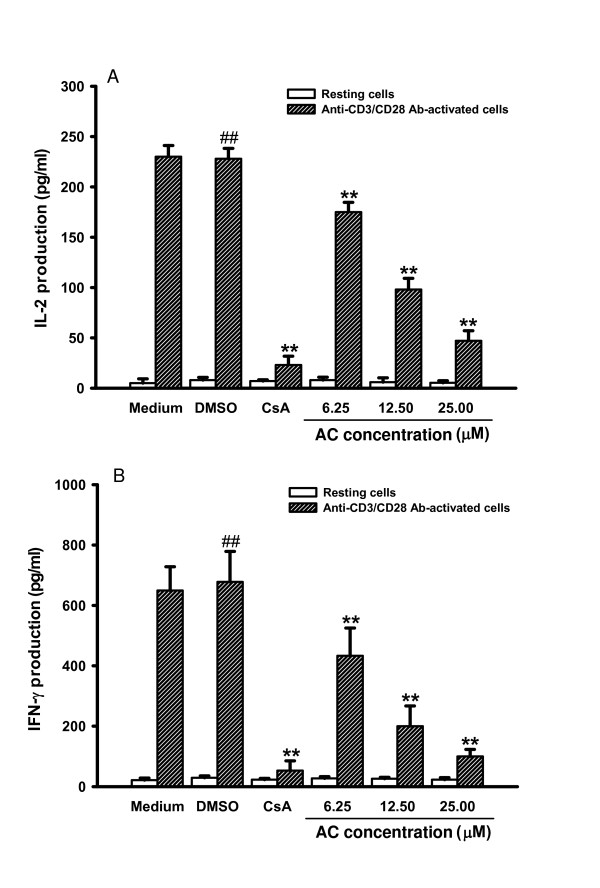
**IL-2 and IFN-γ production in primary human T lymphocytes treated with AC**. Primary human T lymphocytes C (2 × 10^5^/well) were treated by 0, 6.25, 12.5 and 25 μM of AC or CsA (2.5 μM) with or without anti-CD3 (1 μg/ml)/CD28 (3 μg/ml) Ab for three days. Then the cell supernatants were collected and IL-2 and IFN-γ concentrations were determined by EIA, respectively. Each bar is the mean ± SD of three independent experiments. ^## ^*P *< 0.01: vs. the cells treated with DMSO. ** *P *< 0.01: vs. the cells treated with DMSO and anti-CD3/CD28 Ab.

Production of IL-2 and IFN-γ is a hallmark of activated T lymphocytes [16]. To investigate whether AC affected IL-2 and IFN-γ productions in T lymphocytes, we stimulated the cells with anti-CD3/CD28 Ab in the presence or absence of various concentrations of AC (6.25, 12.5 and 25 μM) for three days. Supernatants were then collected and the productions of IL-2 and IFN-γ were determined with EIA. Treatment with anti-CD3/CD28 Ab for three days stimulated IL-2 and IFN-γ productions in primary human T lymphocytes by about 29-fold (*P *= 0.004) and 23-fold (*P *= 0.006) respectively (Figure 3). By contrast, the stimulated production of IL-2 and IFN-γ in activated primary human T lymphocytes was significantly suppressed by 6.25, 12.5 and 25 μM AC (IL-2: *P *= 0.001, *P *= 0.001 and *P *= 0.001 respectively; IFN-γ: *P *= 0.001, *P *= 0.001 and *P *= 0.005 respectively). The inhibitory activity of AC was in a dose-dependent manner (IL-2: *r*=-0.972, *P *= 0.0278; IFN-γ: *r *= -0.936, *P *= 0.0642). AC impaired IL-2 and IFN-γ productions in primary human T lymphoctyes induced by anti-CD3/CD28 Ab.

### Inhibitory effects of AC on IL-2 and IFN-γ gene expression in primary human T lymphocytes

**Figure 4:**
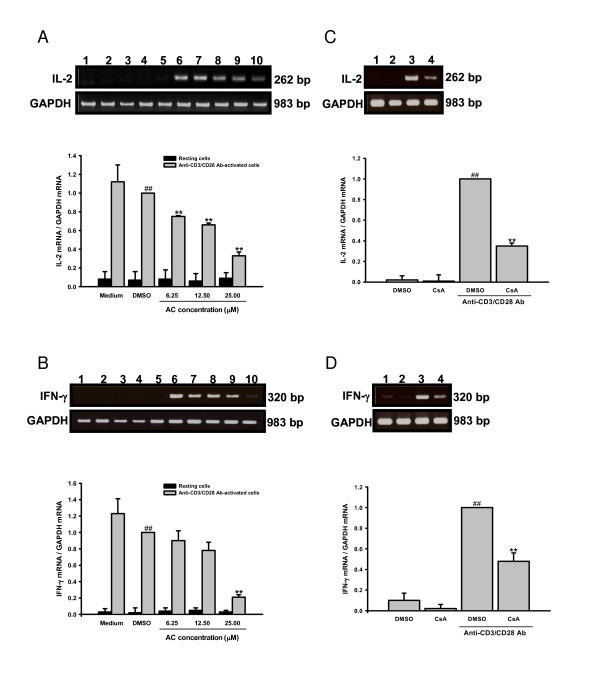
**Effects of AC on IL-2 and IFN-γ transcripts in primary human T lymphocytes induced by anti-CD3/CD28 Ab**. Primary human T lymphocytes (5 × 10^6^) activated with or without anti-CD3 (1 μg/ml)/CD28 (3 μg/ml) Ab in the presence or absence of 6.25, 12.5 or 25 μM AC or CsA (2.5 μM) for 18 hr. The total cellular RNA was isolated from T lymphocytes and aliquots of 1 μg of RNA were reverse-transcribed for synthesis of cDNA. Briefly, 10 μl of cDNA was applied for the PCR test. The PCR was done as described in *Materials and Methods*. After the reaction, the amplified product was taken out of the tubes and run on 2% agarose gel. (A) and (B): Lane 1 - medium, Lane 2 - 0.1% DMSO, Lanes 3 to 5 - 6.25, 12.5 and 25 μM AC, Lane 6 - anti-CD3/CD28 Ab, Lane 7 - DMSO and anti-CD3/CD28 Ab, Lanes 8 to 10 - 6.25, 12.5 or 25 μM AC and anti-CD3/CD28 Ab. (C) and (D): Lane 1 - 0.1% DMSO, Lane 2 - CsA, Lane 3 - DMSO and anti-CD3/CD28 Ab, Lane 4 - CsA and anti-CD3/CD28 Ab. Graphical representation of laser densitometry of IL-2 and IFN-γ mRNA expression in resting or anti-CD3/CD28 Ab-stimulated PBMC in the presence or absence of AC or CsA. Each band was quantitated using laser-scanning densitometer SLR-2D/1D (Biomed Instruments, USA). The ratio of each cytokine mRNA to GAPDH mRNA was calculated. Each bar is the mean ± SD of three independent experiments. ^## ^*P *< 0.01: vs. the cells treated with DMSO. ** *P *< 0.01: vs. the cells treated with DMSO and anti-CD3/CD28 Ab.

To determine whether AC reduced IL-2 and IFN-γ production was related to gene expression, we extracted total cellular RNA from activated primary human T lymphocytes in the presence or absence of AC for 18 hours, ready for RT-PCR. The results of RT-PCR are in Figure 4. The mRNA for GAPDH was detectable in the samples treated with medium (Lane 1), DMSO (0.1%; Lane 2), AC (6.25, 12.5 and 25 μM; Lanes 3 to 5), anti-CD3/CD28 Ab (Lane 6), DMSO and anti-CD3/CD28 Ab (Lane 7), and AC and anti-CD3/CD28 Ab (Lanes 8 to 10) respectively (Figure 4A and 4B). The results indicated that the levels of IL-2 (*P *= 0.003) and IFN-γ (*P *= 0.001) mRNA in T lymphocytes were significantly induced by anti-CD3/CD28 Ab. By contrast, PCR products for both cytokines amplified from activated T lymphocytes RNA preparations were reduced by AC. The laser densitometry analysis demonstrated that the ratio of IL-2 to GAPDH mRNAs in anti-CD3/CD28 Ab-activated T lymphocytes were significantly decreased by 6.25, 12.5 and 25 μM AC (*P *= 0.001, *P *= 0.001 and *P *= 0.001 respectively). AC (25 μM) also significantly ameliorated the ratio of IFN-γ to GAPDH mRNAs in activated T lymphocytes (*P *= 0.001). Thus, AC inhibited IL-2 and IFN-γ production.

### Inhibitory effects of AC on NF-AT activation

**Figure 5:**
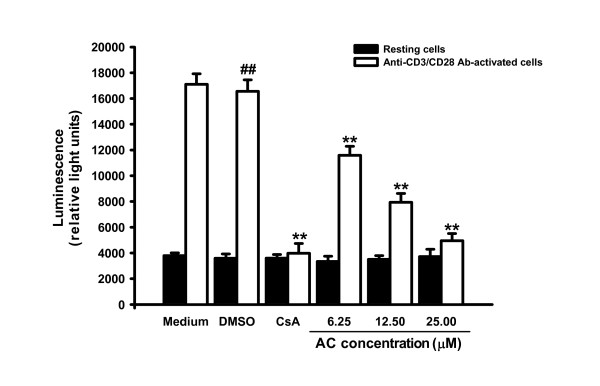
**Effects of AC on NF-AT activation**. Jurkat cells (5 × 10^4^) were transfected with pGL4.30 (luc2P/NFAT-RE/Hygro) by Lipofectamin™ 2000 (Invitrogen, USA) for 24 hours according to the manufacturer's instructions. Then, the cells were cultured with anti-CD3 (1 μg/ml)/CD28 (3 μg/ml) Ab in the presence or absence of AC (6.25, 12.5 and 25 μM) or CsA (2.5 μM) for four hours. Total cell lysates were extracted with 1× reporter lysis buffer (Promega, USA), then 10 μg of total cell lysates were used to determine luciferase activity by the Luciferase Assay System (Promega, USA). Each bar is the mean ± SD of three independent experiments. ^## ^*P *< 0.01: vs. the cells treated with DMSO. ** *P *< 0.01: vs. the cells treated with DMSO and anti-CD3/CD28 Ab.

We used the luciferase assay to determine effects of AC on one major transcription factor, NF-AT, induced by CD3/CD28 signaling and involved in IL-2 and IFN*-γ *gene regulation [17]. The reporter cells, Jurkat cells transfected with pGL4.30 (luc2P/NFAT-RE/Hygro), were cultured in the presence of AC (6.25, 12.5 and 25 μM) for four hours. The cellular proteins were then extracted from the cells and subjected to the luciferase activity assay. As shown in Figure 5, anti-CD3/CD28 Ab induced a 4.6-fold increase in luciferase activity (*P *= 0.001) whereas the vehicle (0.1% DMSO) did not affect this induction. CsA significantly interrupted the luciferase activity in activated T cells (*P *= 0.001). However, treatment with 6.25, 12.5 and 25 μM of AC significantly decreased the luciferase activity of anti-CD3/CD28 Ab-activated cells in a dose-dependent manner (*r *= -0.958, *P *= 0.0418). Thus, AC modulated NF-AT activation.

### Effects of CsA on IL-2, IFN-γ and cell proliferation in T lymphocytes activated with anti-CD3/CD28 Ab

To determine whether AC decreased NF-AT activation, gene expression of IL-2 and IFN-γ and cell proliferation in T lymphocytes, we added CsA (2.5 μM), an NF-AT inhibitor, into T lymphocytes and analyzed gene expression of IL-2 and IFN-γ as well as cell proliferation. While IL-2 (*P *= 0.001) and IFN-γ mRNA (*P *= 0.002) were significantly induced in anti-CD3/CD28 Ab-activated T lymphocytes, CsA signigicantly blocked IL-2 (*P *= 0.001) and IFN-γ (*P *= 0.008) expression in the cells (Figures 4C and 4D). CsA also significantly reduced IL-2 (*P *= 0.001) and IFN-γ (*P *= 0.003) production in the activated cells (Figures 3A and 3B). Furthermore, the T lymphocyte proliferation stimulated by anti-CD3/CD28 Ab was significantly suppressed by CsA (Figure 2A; *P *= 0.003).

## Discussion

Several pharmacological effects were identified in *A. lappa *such as anti-bacterial infection, scavenging free radicals [18], binding platelet-activating factors [19] and inhibiting acute ear swelling [20]. This study showed that AC from *A. lappa *had a profound inhibitory effect on the proliferation of primary human T lymphocytes stimulated by anti-CD3/CD28 Ab. The proliferation-suppressive actions of AC were not explained by a drug-induced reduction in cell viability. We observed that AC decreased production and mRNA expression of IL-2 and IFN-γ and activation of NF-AT in human T lymphocytes induced by anti-CD3/CD28 Ab.

Apart from *A. lappa*, AC is found in various plants such as *Bardanae fructus*, *Saussurea medusa*, *Torreya nucifera *and *lepomea cairica*. AC prevents leukocytes from recruitment into the inflamed tissue [21]. AC blocks TNF-α production by impairments of AP-1 activation [9]. The present study demonstrated that AC suppressed proliferation and IL-2 and IFN-γ production in primary human T lymphocytes activated by anti-CD3/CD28 Ab. AC is a potent inducer of apoptosis for HL-60 T leukemia cells, MH60 B lymphoma cells and SW480 colon cancer cells [22]. Thus, we could not rule out the possibility that AC inhibited the proliferation of primary human T lymphocytes *via *the apoptosis pathway. The possible inhibitory effect of DMSO on primary human T lymphocytes was also studied in these experiments. The cell proliferation and viability were not changed by DMSO. Therefore, the inhibitory function of AC was unlikely related to DMSO.

Interaction of T lymphocytes with antigens initiates a cascade of genes expression such as IL-2 and IFN-γ mRNA inducing the resting T cells to proliferate [23]. This study showed that AC inhibited IL-2 and IFN-γ productions in primary human T lymphocytes stimulated by anti-CD3/CD28 Ab. The impairments of IL-2 and IFN-γ production were related to the suppression of their mRNA transcriptions by AC. Since T lymphocyte proliferation is primarily mediated by IL-2, inhibition of IL-2 production is a central mechanism of action of several immunosuppressants such as CsA. This study also demonstrated that CsA inhibited IL-2 and IFN-γ gene expression and cell proliferation in primary human T lymphocytes induced by anti-CD3/CD28 Ab, suggesting that AC actions are similar to those of CsA which induces arrest activation and proliferation of T cells by inhibiting IL-2 transcription [14]. Furthermore, the preliminary data from immunofluorescence staining indicated that AC had no effect on IL-2 receptor expression in primary human T lymphocytes activated by anti-CD3/CD28 Ab (data not shown), suggesting that the reduction of proliferation in AC-treated T lymphocytes was not caused by down-regulation of IL-2 receptor expression. Failure to produce IL-2 and IFN-γ may be the reason why primary human T lymphocytes do not proliferate.

NF-AT is a major player in the control of T lymphocytes activation and proliferation [2]. After anti-CD3/CD28 Ab stimulation, calcium-dependent phosphatase calcineurin binds to NF-AT, dephosphorylates NF-AT and causes nuclear import of NF-AT. The binding domain of NF-AT is Rel similarity domain located in numerous cytokine promoters. IL-2 and IFN-γ gene expressions in T lymphocytes are controlled by NF-AT-dependent promoters/enhancers [24]. This study found that AC decreased NF-AT activation. NF-AT is a target for the immunosuppressants CsA and FK506 which are efficient inhibitors of T cell activation [14]. This study also demonstrated that CsA blocked NF-AT activation, suggesting that AC inhibited IL-2 and IFN-γ production and cell proliferation in primary human T lymphocytes by modulation of NF-AT activation. Interleukin-10 is mainly produced by regulatory T lymphocytes and regulates other immune cells [24]. We also showed that AC (25 μM) did not affect IL-10 production in primary human T lymphocytes induced by anti-CD3/CD28 Ab (453 ± 88 vs. 412 ± 75pg/ml).

## Conclusion

AC inhibited T lymphocyte proliferation and decreased the gene expression of IL-2, IFN-γ and NF-AT.

## Abbreviations

Ab: antibody; AC: arctigenin; RT-PCR: reverse transcription-polymerase chain reaction; IL-2: interleukin-2; IFN-γ: interferon-γ; PBMC: peripheral blood mononuclear cells; EtOAc: ethyl acetate; DMSO: dimethylsulfoxide; PBS: phosphate buffered saline; HBSS: Hanks' buffer saline solution; FCS: fetal calf serum; CsA: cyclosporin A; NF-AT: nuclear factor of activated T cells; EIA: enzyme immunoassays; DEPC: diethyl pyrocarbonate; EDTA: ethylenediaminetetraacetate; DTT: dithiothreitol; GAPDH: glyceraldehyde-3-phosphate dehydrogenase

## Competing interests

The authors declare that they have no competing interests.

## Authors' contributions

WJT and CTC designed and conducted the experiments. GJW and THL isolated and purified AC from *A. lappa*. SFC and SCL constructed pGL4.30 (luc2P/NFAT-RE/Hygro) plasmids. YCK supervised the study and revised the manuscript. All authors read and approved the final version of the manuscript.
